# Effects of arginine vasopressin on the transcriptome of prefrontal cortex in autistic rat model

**DOI:** 10.1111/jcmm.17578

**Published:** 2022-10-14

**Authors:** Bo Zhou, Xuehui Yan, Liu Yang, Xiaoli Zheng, Yunhua Chen, Yibu Liu, Yibing Ren, Jingang Peng, Yi Zhang, Jiayu Huang, Lei Tang, Min Wen

**Affiliations:** ^1^ State Key Laboratory of Functions and Applications of Medicinal Plants Guizhou Medical University Guizhou China; ^2^ Guizhou Provincial Engineering Technology Research Center for Chemical Drug R&D Guizhou Medical University Guizhou China; ^3^ College of Pharmacy Guizhou Medical University Guizhou China; ^4^ Department of Neurology Wuhan Third Hospital (Tongren Hospital of Wuhan University) Wuhan China; ^5^ College of Basic Medical Guizhou Medical University Guizhou China

**Keywords:** arginine vasopressin, autism spectrum disorder, neurodevelopmental, oligodendrocyte, prefrontal cortex, synapse

## Abstract

Our previous studies have also demonstrated that AVP can significantly improve social interaction disorders and stereotypical behaviours in rats with VPA‐induced autism model. To further explore the mechanisms of action of AVP, we compared the PFC transcriptome changes before and after AVP treatment in VPA‐induced autism rat model. The autism model was induced by intraperitoneally injected with VPA at embryonic day 12.5 and randomly assigned to two groups: the VPA‐induced autism model group and the AVP treatment group. The AVP treatment group were treated with intranasal AVP at postnatal day 21 and for 3 weeks. The gene expression levels and function changes on the prefrontal cortex were measured by RNA‐seq and bioinformatics analysis at PND42 and the mRNA expression levels of synaptic and myelin development related genes were validated by qPCR. Our results confirmed that AVP could significantly improve synaptic and axon dysplasia and promote oligodendrocyte development in the prefrontal cortex in VPA‐induced autism models by regulating multiple signalling pathways.

## 1 INTRODUCTION

Autism spectrum disorder (ASD) refers to a series of neurodevelopmental diseases characterized by two hallmark symptoms, social communication deficits and repetitive behaviours.^1^ The prevalence of ASD has been steadily increasing over the past two decades, with current estimates reaching up to 23.0 per 1000 (1 in 44) children aged 8 years, and ASD was 4.2 times as prevalent among boys as among girls.^2^ Although many researches have been carried out, the precise molecular mechanisms of ASD are still not fully elucidated and no medications are currently approved for ameliorating ASD's core social behaviour deficits.^3^, ^4^

Prefrontal cortex (PFC), which serves as the chief executive officer of the brain, maintains diverse connections with other cortical and sub‐cortical regions of the brain, including hub regions of ‘the social brain’ such as the nucleus accumbens (NAc), amygdala, ventral tegmental area (VTA), hypothalamus, and regions of the cortex involved in processing sensory and motor inputs, responsible for information processing that subserve cognitive, emotional and social behaviour.^5^, ^6^, ^7^, ^8^ Multiple lines of evidence suggest that PFC structural and functional abnormalities are strongly associated with social malfunctioning in ASD. Pagani et al.^9^ found that mutations in the synaptic scaffolding protein SHANK3 may disrupt the connectivity of prefrontal areas and underpin socio‐communicative impairments in autism. Bertero et al.^10^ found that autism‐associated chromosome 16p11.2 microdeletion may impair prefrontal functional connectivity in mouse and human, resulting in weaker long‐range functional coupling with temporal–parietal regions and socio‐cognitive impairments. Yizhar et al.^11^, ^12^, ^13^ found that disturbed the balance of excitation and inhibition (E/I balance) in synaptic transmission and neural circuits in PFC, leads to specific social dysfunction, and modulation of prefrontal cortex excitation/inhibition balance rescues social behaviour.^14^

Recent studies have shown that arginine vasopressin can significantly improve social dysfunction in autistic patients, but the precise mechanism remains unclear.^15^, ^16^ Our previous studies have also demonstrated that AVP can significantly improve social interaction disorders and stereotypical behaviours in rats with VPA‐induced autism model.^17^ To further explore the mechanisms of action of AVP, we compared the PFC transcriptome changes before and after AVP treatment in VPA‐induced autism rat model.

## 2 MATERIALS AND METHODS

### 2.1 Materials

#### 2.1.1 Chemicals

Valproic acid sodium salt (P4543‐10G) was purchased from Sigma‐Aldrich Co. (St Louis, MO, USA). Argipressin (AVP, HY‐P0049) was purchased from MedChemExpress LLC.

#### 2.1.2 Animals

Male and female Wistar rats (270–290 g) were obtained from the Department of Experimental Animal Center of Guizhou Medical University. Animals were housed individually with water and chow freely available under a regulated environment (23 ± 2°C; 50% ± 10% humidity) with a 12/12 h light–dark cycle. All experiments were approved by Guizhou Medical University Animal Care Welfare Committee.

### 2.2 Methods

#### 2.2.1 Animal model

As previously described,^18^ female and male rats were allowed to mate overnight and the morning when spermatozoa were found was designated as embryonic day 0.5 (E0.5). The pregnant rats were randomly distributed into two groups: VPA group (*n* = 10) and saline group (*n* = 5). On E12.5, the VPA group were intraperitoneally injected with sodium valproate (dose of 600 mg/kg, 250 mg/ml dissolved in physiological saline); the saline groups were received the same volume of normal saline at same time. The day of birth of the offspring was marked as postnatal day 1 (PND 1). After weaning at postnatal day 21 (PND21), offspring of same sex were housed separately with 4–5 per cage. To assess the treatment effects of AVP, the offspring of VPA group were randomly divided into two groups: autism groups (*n* = 10) and AVP treatment group (*n* = 10). AVP treatment group received a daily intranasal of AVP (dose of 400 μg/kg, 2.5 mg/ml dissolved in NS) from PND21 to PND42. The offspring of saline groups were marked as control group. The control groups and autism group were given the same amount of saline. All experiments were carried out on male offspring.

#### 2.2.2 RNA isolation

Five rats in each group were sacrificed at P42 and the prefrontal cortex was quickly dissected and frozen in liquid nitrogen for 2 h, thereafter stored at −80°C until used. Total RNA was extracted and purified using TRIzol reagent (Invitrogen) following the manufacturer's procedure. The RNA concentration and purity of each sample was quantified using NanoDrop ND‐1000 (NanoDrop). RNA quality was measured by Bioanalyzer 2100 (Agilent). All RNA samples included in the expression analysis had an A260/A280 absorbance ratio greater than 1.8 and RNA integrity number (RIN) > 7.0.

#### 2.2.3 RNA‐seq

An RNA‐seq library of each sample was prepared and performed the 2 × 150 bp paired‐end sequencing (PE150) on an Illumina Novaseq™ 6000 (LC‐Bio Technology Co., Ltd.) following the vendor's recommended protocol.

#### 2.2.4 Sequence analysis

##### Differentially expressed genes analysis

Raw data files in FASTQ format were generated from the Illumina Novaseq™ 6000. The reads after quality control and preprocessing were aligned to the rattus_norvegicus reference genome (v101) using the HISAT2^19^ and the gene expression counts were derived using HTSEQ.^20^ The differentially expressed genes were selected with fold changes >1.3 and FDR < 0.05 by DESeq2 package.^21^ The Gene Ontology (GO) and Kyoto Encyclopedia of Genes and Genomes (KEGG) enrichments of the differentially expressed genes were performed by the Bioconductor ‘clusterProfiler’ package (FDR < 0.05 and *p*.adj < 0.05).^22^, ^23^ The Enrichment Map which displayed the gene overlap relationship between the enriched GO terms were performed by the Bioconductor ‘enrichplot’ package, and the analysis results were visualized by the R package ‘ggplot2’. Synapse function and gene enrichment studies were performed by the on‐line database Synaptic Gene Ontologies (SynGO, https://syngoportal.org/), a public knowledgebase for synapse research.^24^

##### Gene set enrichment analysis (GSEA)

Traditional GO/KEGG analysis strategies usually focus on a handful of differential expression gene. Although useful, they are easily affected by the filter threshold (logFC and FDR) and lead to miss some genes with moderate differential expression but important biological significance. To overcome many genes with moderate but meaningful expression, changes are discarded by the strict cut‐off value, which leads to a reduction in statistical power, we conducted a Gene Set Enrichment Analysis (GSEA).^25^ Gene Set Enrichment Analysis were used to identify statistically enriched gene sets in transcriptomic data by GSEA V4.2.3 with |NES| > 1 and with NOM *p*‐value < 0.05 and FDR < 0.25.^25^, ^26^ The biological process (c5.go.bp.v7.5.1) and KEGG gene sets (c2.cp.kegg.v7.5.1) were annotation by Molecular Signatures Database v7.5.1.^27^, ^28^

##### Expression patterns analysis

Soft clustering was performed using Mfuzz^29^ to mine the expression patterns of top 3000 most variable genes across all samples. In the clustering analysis, the optimal cluster number was calculated using default parameters. Clusters with different change trends of genes were subsequent functional annotation analysis by g:Profiler^30^ (https://biit.cs.ut.ee/gprofiler/gost).

### 2.3 qRT‐PCR confirmation

Total RNA of prefrontal cortex was extracted utilizing TRIzol reagent (Invitrogen) and reverse transcribed to cDNA using the Thermo Scientific Revert Aid First Strand cDNA Synthesis Kit (Thermo Fisher Scientific Inc.). The relative expression levels of the target gene were calculated using the 2^−ΔΔCt^ method and normalized to GAPDH.^31^.The primer sequences used are listed in Table 1.

**TABLE 1:** The primer sequences list

Gene	Primer	Sequence (5′–3′)	PCR Products
Rat GAPDH	Forward	ACAGCAACAGGGTGGTGGAC	253 bp
	Reverse	TTTGAGGGTGCAGCGAACTT	
Rat Syn1	Forward	TTCTCCTCGCTGTCTAACGC	182 bp
	Reverse	CCATGGATCTTCTTCCCTTT	
Rat Synpo	Forward	AGACGCCCCTTAGGAAACT	231 bp
	Reverse	CTCCACGAACGTGAACATT	
RAT Lingo1	Forward	CAGGGCAAGGAGTTCAAGGAC	179 bp
	Reverse	CGGGGTGAGAGCCAAAGGATA	
Rat Chd7	Forward	CCTCCATCATCCTTCCCCTAACCA	220 bp
	Reverse	ATAGACTCCCATCTGACCGCCACC	
Rat Olig2	Forward	TGAAGAGACTGGTGAGCGAG	165 bp
	Reverse	GAGGGAGGATGGGGTGATG	
Rat MBP	Forward	ATGTGTTTGGGGAGGCAGAT	233 bp
	Reverse	TTGGATGGTCTGAAGCTCGT	

### 2.4 Statistical analysis

All qPCR data are represented as the mean ± SD. Inter‐group statistical significance was determined by Student's *t*‐test using STATA 14.0 (StataCorp). Statistical significance was set at *p* < 0.05.

## 3 RESULTS

### 3.1 Quality control and reads mapping

The number of total reads, clean reads, the sequencing error rate, the percentage of Q20, Q30 and the GC content of all samples meet the quality control requirements of sequencing. Over 96.34% of the clean reads were mapped to the rat genome, and over 76.65% of them were mapped to a single genome location.

### 3.2 Differentially expressed analysis

#### 3.2.1 Control group compared with VPA‐induced autism model group

**FIGURE 1:**
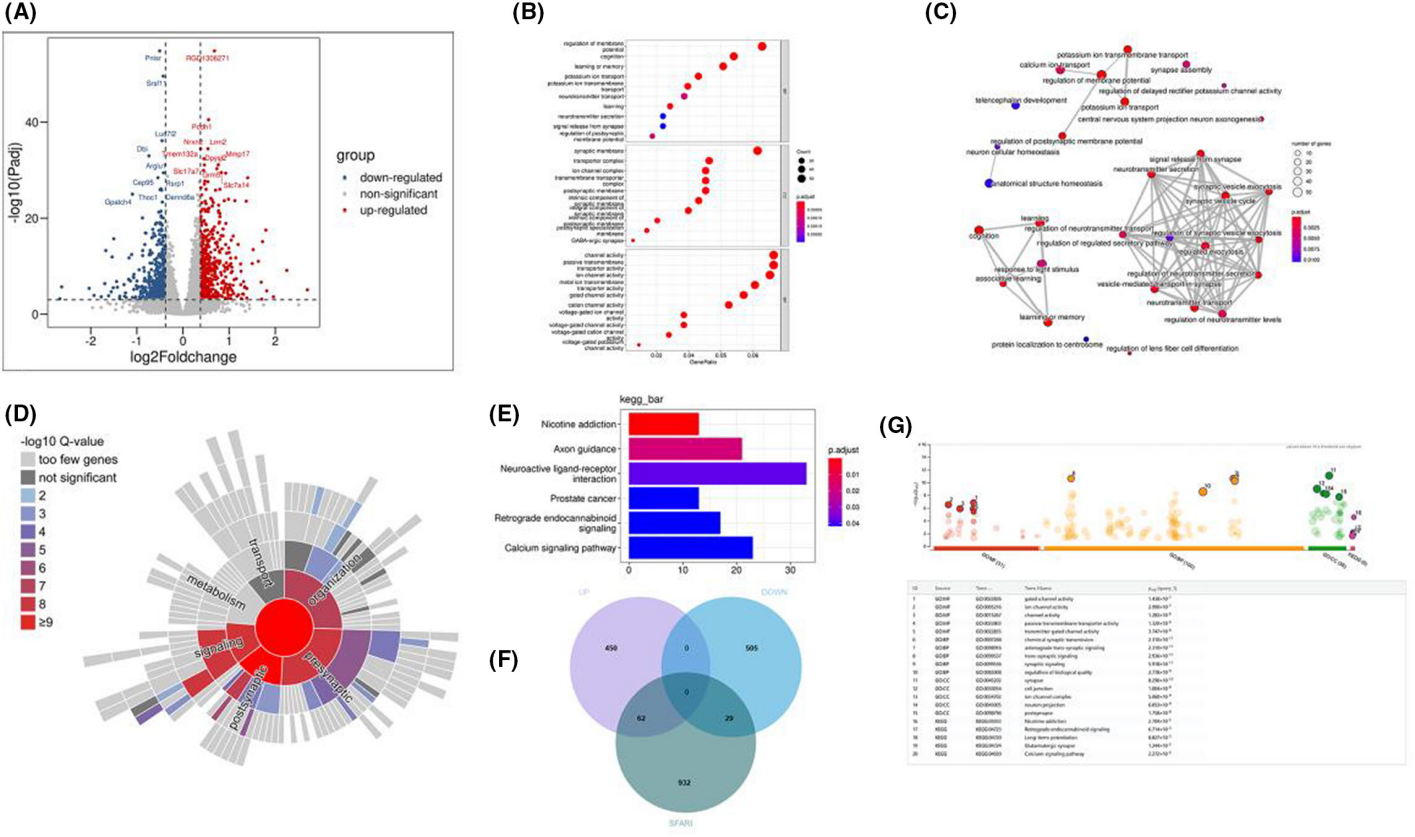
Functional analysis of differential expressed genes between normal group and model group. (A) Volcano plot showing differentially expressed genes in PFC. (B) Dot plot showing GO enrichment analysis of differentially expressed genes in PFC. (C) Enrichment map organizes enriched GO BP terms into a network with edges connecting overlapping genes. Mutually overlapping genes tend to cluster together, making it easy to identify functional module. (D) Sunburst plot representing biological processes enrichment analysis of upregulated synaptic genes in PFC. Higher red intensities are associated with more significant enrichments. All the identified synaptic genes are represented in the red circle at the centre of the plot. (E) Bar Plot showing KEGG enrichment result about differentially expressed genes in PFC. (F) Venn diagram showing the overlapping of downregulated (DOWN) genes, upregulated (UP) genes and the genetic association for ASD (SFARI). (G) Function annotation of genes which overlapping of downregulated (DOWN) genes, upregulated (UP) genes and the genes of genetic association for ASD (SFARI).

A total of 1046 genes (512 up and 534 down) was significantly different in the VPA‐induced autism model group compared with control group (Volcano plot with the differentially expressed genes were show in Figure 1A). To identify the most biologically relevant functions of these differential expression genes (DEGs), we performed GO functional enrichment analyses. Our results show that most of these DEGs are located in the synaptic membrane and have channel activity, which is involved in cognition, learning and memory, the release and transmission of neural signals and so on (*p*.adjust < 0.05, Figure 1B). The results of enrichment map showed that most GO terms were enriched in synaptic signalling (Figure 1C). Clearly, the VPA‐induced neurodevelopmental disorders are associated with disturbances in synaptic signalling. Given the enrichment of GO pathways involved in synaptic function for DEGs, we further analysis them with SynGO (https://syngoportal.org/). 109 of these upregulated DEGs were identified as synaptic genes. Synaptic enrichment analyses identified 21 Cellular Component and 28 biological processes terms are significantly enriched (1% FDR, testing terms with at least three matching input genes), with presynapse, postsynapse, postsynaptic specialization, postsynaptic density membrane and synaptic vesicle as the most enriched in subcellular components, and the process in the postsynapse, process in the presynapse, synapse organization, synaptic signalling and trans‐synaptic signalling as the most enriched in biological processes (Figure 1D). On the contrary, no enriched synaptic components or processes were present with downregulated DEGs. In addition, the KEGG pathway enrichment analysis showed that these DEGs were significantly enriched in 6 pathways, such as Nicotine addiction, Axon guidance, Neuroactive ligand‐receptor interaction, Prostate cancer, Retrograde endocannabinoid signalling and Calcium signalling pathway (Figure 1E). Strikingly, we also analysed the intersection between our differential expression gene list and the list of human gene which have evidence of genetic association with ASD from the SFARI (https://gene.sfari.org/database/human‐gene/). Ninety‐one differential expression genes were found in the SFARI database, which are mainly involved in anterograde trans‐synaptic signalling, chemical synaptic transmission, trans‐synaptic signalling, synaptic signalling and nervous system development and so on (Figure 1F,G).

#### 3.2.2 VPA‐induced autism model group compared with AVP group

**FIGURE 2:**
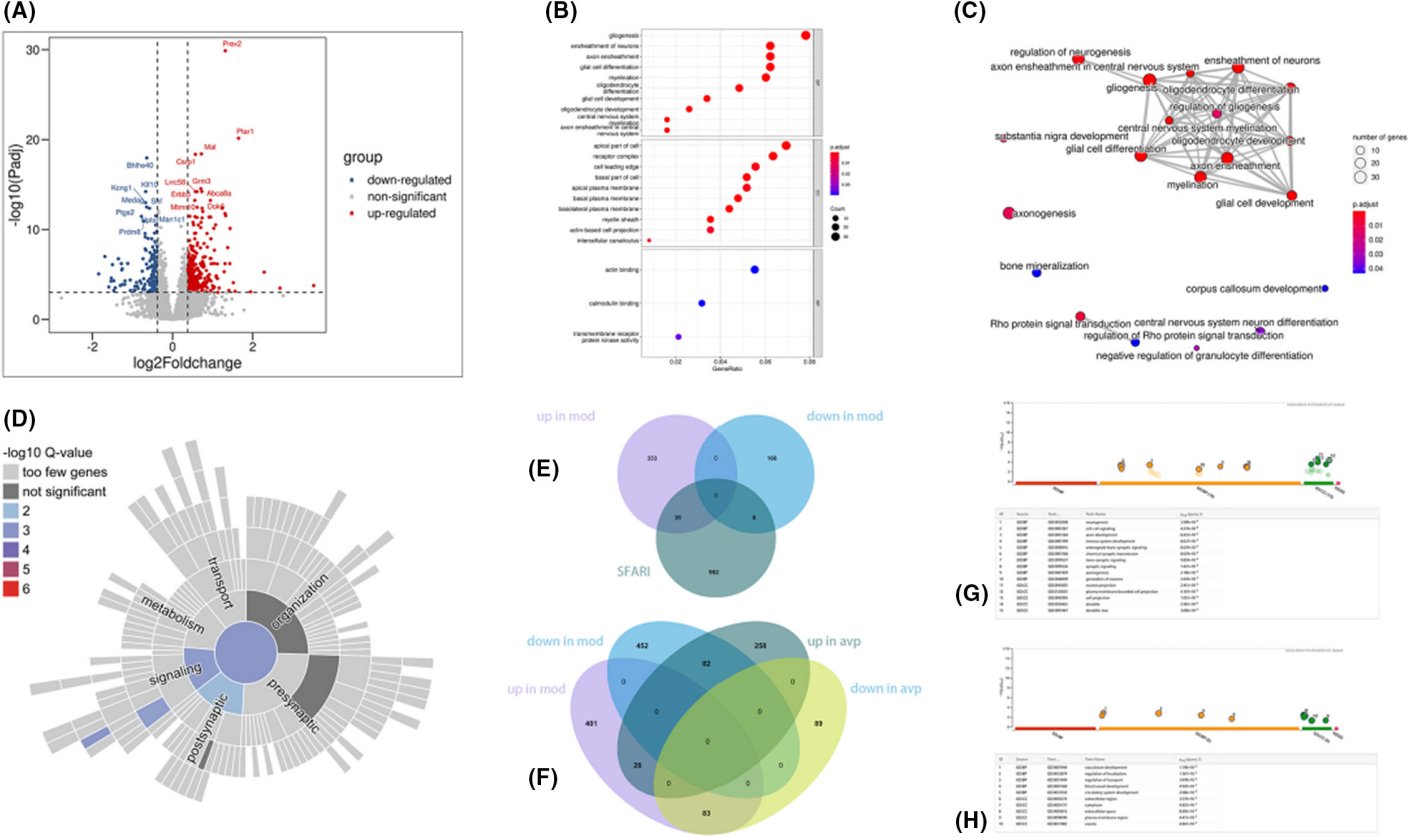
Functional analysis of differential genes between model group and AVP group. (A) Volcano plot showing differentially expressed genes in PFC. (B) Dot plot showing GO enrichment analysis of differentially expressed genes in PFC. (C) Enrichment map organizes enriched GO BP terms into a network with edges connecting overlapping genes. Mutually overlapping genes tend to cluster together, making it easy to identify functional module. (D) Sunburst plot representing biological processes enrichment analysis of upregulated synaptic genes in PFC. (E) Venn diagram showing the overlapping of downregulated (DOWN) genes, upregulated (UP) genes and the genetic association for ASD (SFARI). (F) Venn diagram showing the overlapping of downregulated (DOWN) genes and upregulated (UP) genes between normal, model and AVP groups. (G) Function annotation of genes which upregulated in model group and downregulated in AVP group. (H) Function annotation of genes which downregulated in model group and upregulated in AVP group.

A total of 540 genes (368 up and 172 down) were significantly different in the VPA‐induced autism model group compared with AVP group (the Volcano plot showing differentially expressed genes in PFC were show in Figure 2A). GO functional enrichment analyses showed that most of these DEGs are involved in gliogenesis, glial cell differentiation and development, oligodendrocyte differentiation and development, axon ensheathment and myelination and so on (*p*.adjust < 0.05, Figure 2B). The same result was displayed in Enrichment Map (Figure 2C). Clearly, AVP promotes oligodendrocyte development of prefrontal cortex, which is consistent with our findings in the amygdala.^17^ No KEGG signalling pathway was enriched with all differential expression genes. To further analyse whether AVP treatment affects synaptic development, synGO was also used for enrichment analysis of DEGs. Twenty‐five of these downregulated DEGs were identified as synaptic genes. Synaptic enrichment analyses identified 0 Cellular Component and 5 biological processes terms are significantly enriched (1% FDR, testing terms with at least three matching input genes). The biological processes are concentrated in process in synaptic signalling, chemical synaptic transmission, process in the postsynapse, postsynaptic modulation of chemical synaptic transmission (Figure 2D). We also analysed the intersection between our differential expression gene list and the list of SFARI human gene. Of 540 DEGs, 41 genes were found in the SFARI database (Figure 2E). Thirty‐five upregulated gene were mainly involved in development such as anatomical structure development, multicellular organismal process, system development, developmental process, nervous system development, and 6 downregulated genes were mainly involved in behaviour, chemical synaptic transmission, anterograde trans‐synaptic signalling, trans‐synaptic signalling and synaptic signalling. In addition, we also performed Venn diagram analysis of differential genes between normal, autism model and AVP groups (Figure 2F). Our results showed that 83 genes which up in model group and down in avp group were mainly involved in neurogenesis, nervous system development, axon development, synaptic signalling and so on (Figure 2G), and 82 genes which down in model group and up in AVP group were mainly involved in vasculature development, regulation of localization, regulation of transport, blood vessel development and circulatory system development (Figure 2H).

### 3.3 Gene set enrichment analysis (GSEA)

**FIGURE 3:**
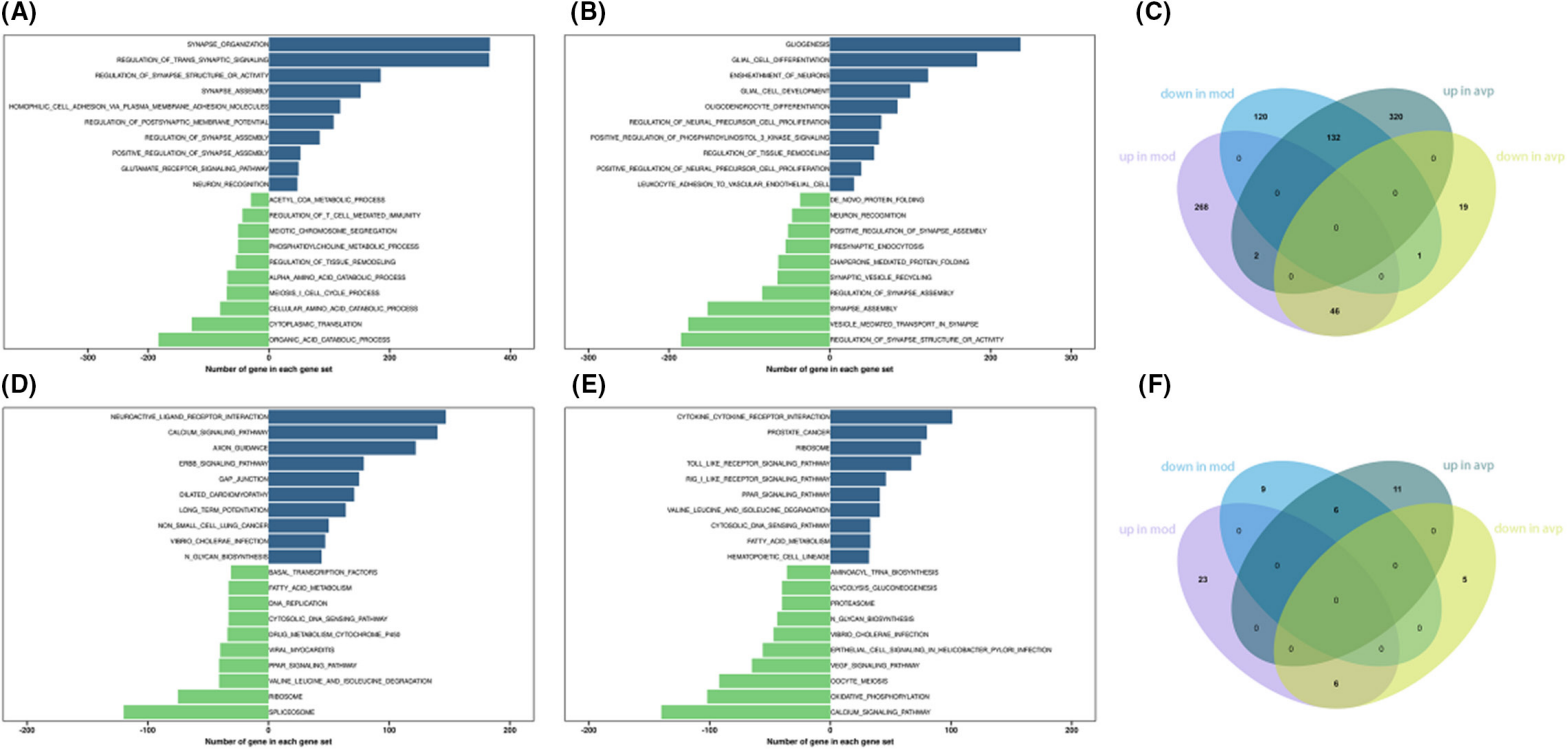
The results of Gene set enrichment analysis. (A) Differentially expressed of GO BP gene sets in PFC between control and model group. (B) Differentially expressed of GO BP gene sets in PFC between model and avp group. (C) Venn diagram showing the overlapping of downregulated (DOWN) genes, upregulated (UP) GO gene sets between normal, model and AVP groups. (D) Differentially expressed of KEGG gene sets in PFC between control and model group. (E) Differentially expressed of KEGG gene sets in PFC between model and avp group. (F) Venn diagram showing the overlapping of downregulated (DOWN) genes, upregulated (UP) KEGG gene sets between normal, model and AVP groups.

Compared with control group, 316 GO BP gene sets are significantly upregulated in VPA‐induced autism model group. The top 3 terms were significantly enriched in synapse organization, regulation of trans‐synaptic signalling and regulation of synapse structure or activity. 253 GO BP gene sets are significantly downregulated in VPA‐induced autism model group, the top 3 terms were significantly enriched in organic acid catabolic process, cytoplasmic translation and cellular amino acid catabolic process (Figure 3A). In the VPA‐induced autism model group compared with AVP treatment group, 66 GO BP gene sets are significantly downregulated in AVP treatment group. The top 3 terms were significantly enriched in regulation of synapse structure or activity, vesicle mediated transport in synapse and synapse assembly. 454 GO BP gene sets are significantly upregulated in AVP treatment group. The top 3 terms were significantly enriched in gliogenesis, glial cell differentiation and ensheathment of neurons (Figure 3B).

Compared with control group, 28 KEGG gene sets are significantly upregulated in VPA‐induced autism model. The top 3 terms were significantly enriched in neuroactive ligand–receptor interaction, calcium signalling pathway and axon guidance. Fifteen KEGG gene sets were significantly downregulated in VPA‐induced autism model group. The top 3 terms were significantly enriched in spliceosome, ribosome and valine leucine and isoleucine degradation (Figure 3D).

Compared with VPA‐induced autism model group, 11 gene sets are significantly downregulated in avp treatment group. The top 3 terms were significantly enriched in calcium signalling pathway, oxidative phosphorylation and oocyte meiosis. Seventeen gene sets are significantly upregulated in avp treatment group. The top 3 KEGG pathway were significantly enriched in cytokine–cytokine receptor interaction, prostate cancer and ribosome (Figure 3E).

**FIGURE 4:**
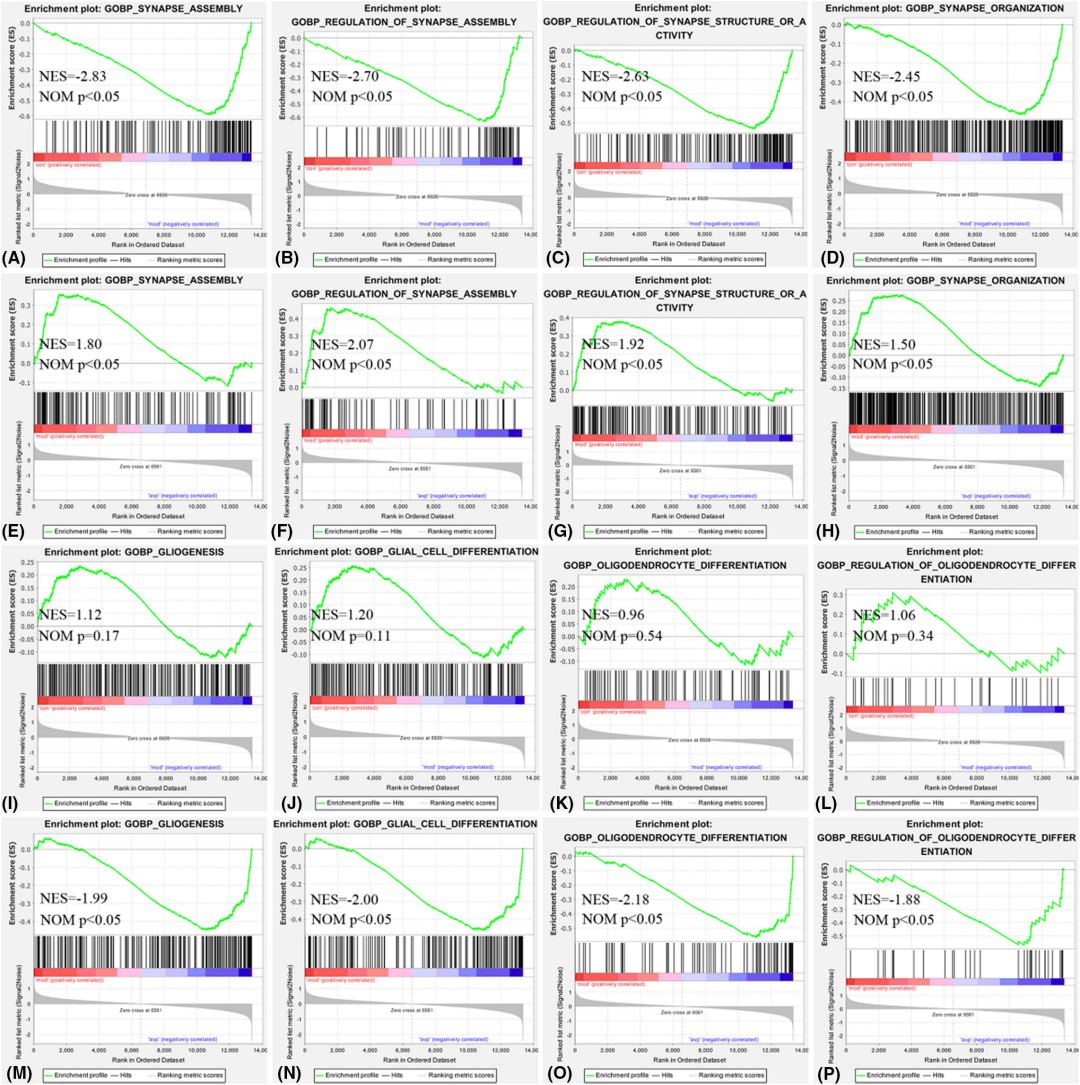
Enrichment of related biological process by GSEA. (A, E) Synapse assembly in control (con) vs. autism (mod) and autism (mod) vs. AVP, (B, F) Regulation of synapse assembly in control (con) vs. autism (mod) and autism (mod) vs. AVP, (C, G) Regulation of synapse structure or activity in control (con) vs. autism (mod) and autism (mod) vs. AVP, (D, H) Synapse organization in control (con) vs. autism (mod) and autism (mod) vs. AVP, (I, M) Gliogenesis in control (con) vs. autism (mod) and autism (mod) vs. AVP, (J, N) Glial cell differentiation in control (con) vs. autism (mod) and autism (mod) vs. AVP, (K, O) Oligodendrocyte differentiation in control (con) vs. autism (mod) and autism (mod) vs. AVP, (L, P) Regulation of oligodendrocyte differentiation in control (con) vs. autism (mod) and autism (mod) vs. AVP.

The intersection of the gene sets variation between VPA‐induced autism model group (con vs mod) and AVP group (mod vs AVP) were analysis by Evenn (http://www.ehbio.com/test/venn/#/). 132 BP gene sets were remarkable downregulated in VPA‐induced autism model group and upregulate in AVP treatment, such as acetyl‐CoA metabolic process, phosphatidylcholine metabolic process, alpha amino acid catabolic process, regulation of T cell‐mediated immunity, organic acid catabolic process and so on. Forty‐six BP gene sets were remarkable upregulated in VPA‐induced autism model group and downregulate in AVP treatment, such as synapse assembly, regulation of synapse assembly, regulation of synapse structure or activity, positive regulation of synapse assembly and regulation of postsynaptic membrane potential and so on. Furthermore, some glial cell development‐related BP such as oligodendrocyte differentiation, oligodendrocyte development, glial cell differentiation, glial cell development, gliogenesis and so on were not change in VPA‐induced autism model group but remarkable up regulated after AVP treatment (Figures 3C and 4A–P).

**FIGURE 5:**
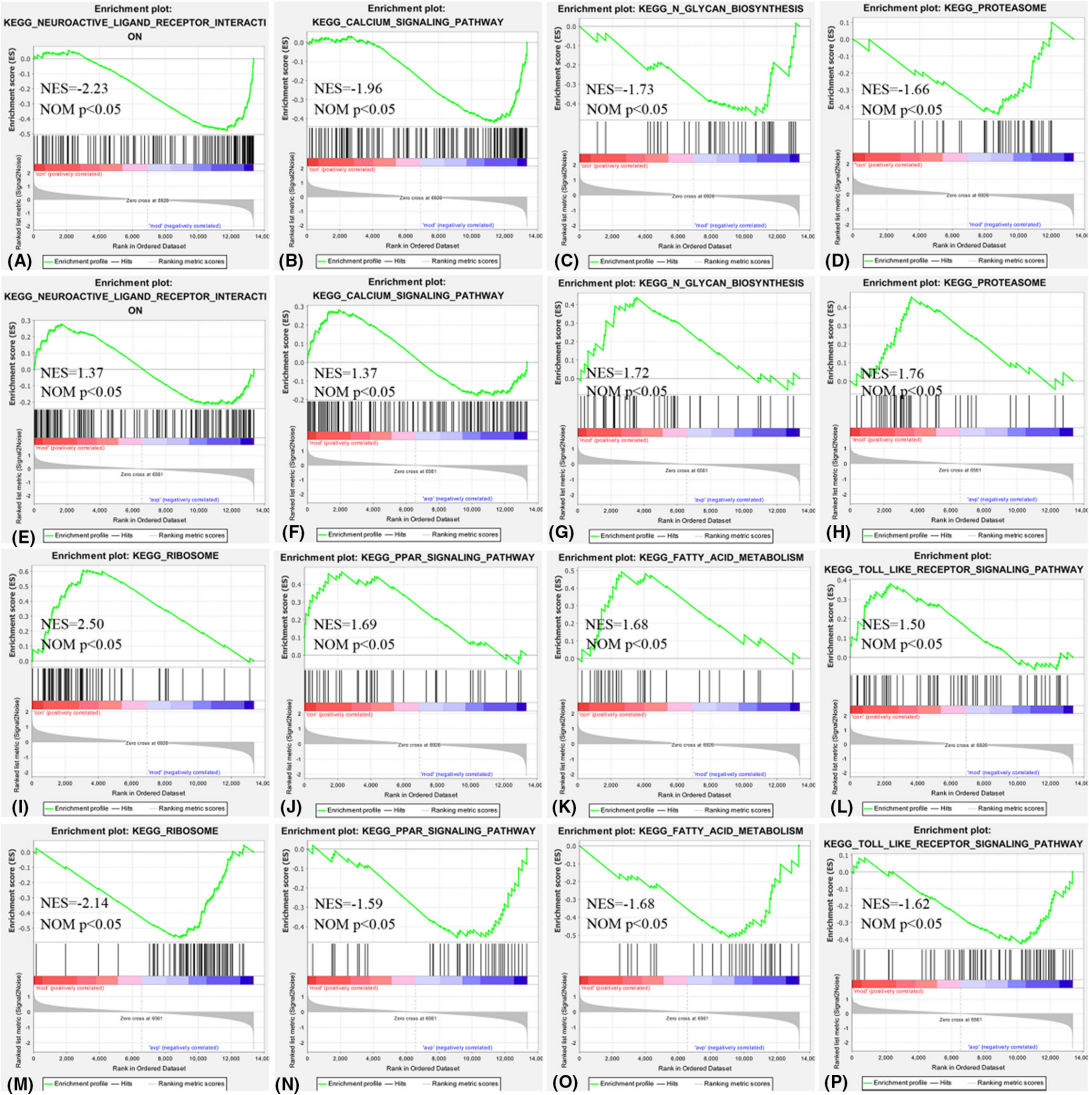
Enrichment of related kegg by GSEA. (A, E) Neuroactive ligand–receptor interaction in control (con) vs. autism (mod) and autism (mod) vs. AVP, (B, F) Calcium signalling pathway in control (con) vs. autism (mod) and autism (mod) vs. AVP, (C, G) N‐glycan biosynthesis in control (con) vs. autism (mod) and autism (mod) vs. AVP, (D, H) Proteasome in control (con) vs. autism (mod) and autism (mod) vs. AVP, (I, M) Ribosome in control (con) vs. autism (mod) and autism (mod) vs. AVP, (J, N) PPAR signalling pathway in control (con) vs. autism (mod) and autism (mod) vs. AVP, (K, O) Fatty acid metabolism in control (con) vs. autism (mod) and autism (mod) vs. AVP, (L, P) Toll like receptor signalling pathway in control (con) vs. autism (mod) and autism (mod) vs. AVP.

Six KEGG gene sets was found remarkable downregulated in VPA‐induced autism model group and upregulate in AVP treatment, such as ribosome, cytosolic DNA sensing pathway, valine leucine and isoleucine degradation, fatty acid metabolism, PPAR signalling pathway and Toll‐like receptor signalling pathway. Six KEGG gene sets were found remarkable upregulated in VPA‐induced autism model group and downregulate in AVP treatment, such as neuroactive ligand–receptor interaction, vibrio cholerae infection, calcium signalling pathway, N‐glycan biosynthesis, proteasome and oocyte meiosis (Figures 3F and 5A–P).

### 3.4 Dynamics of genes expression

**FIGURE 6:**
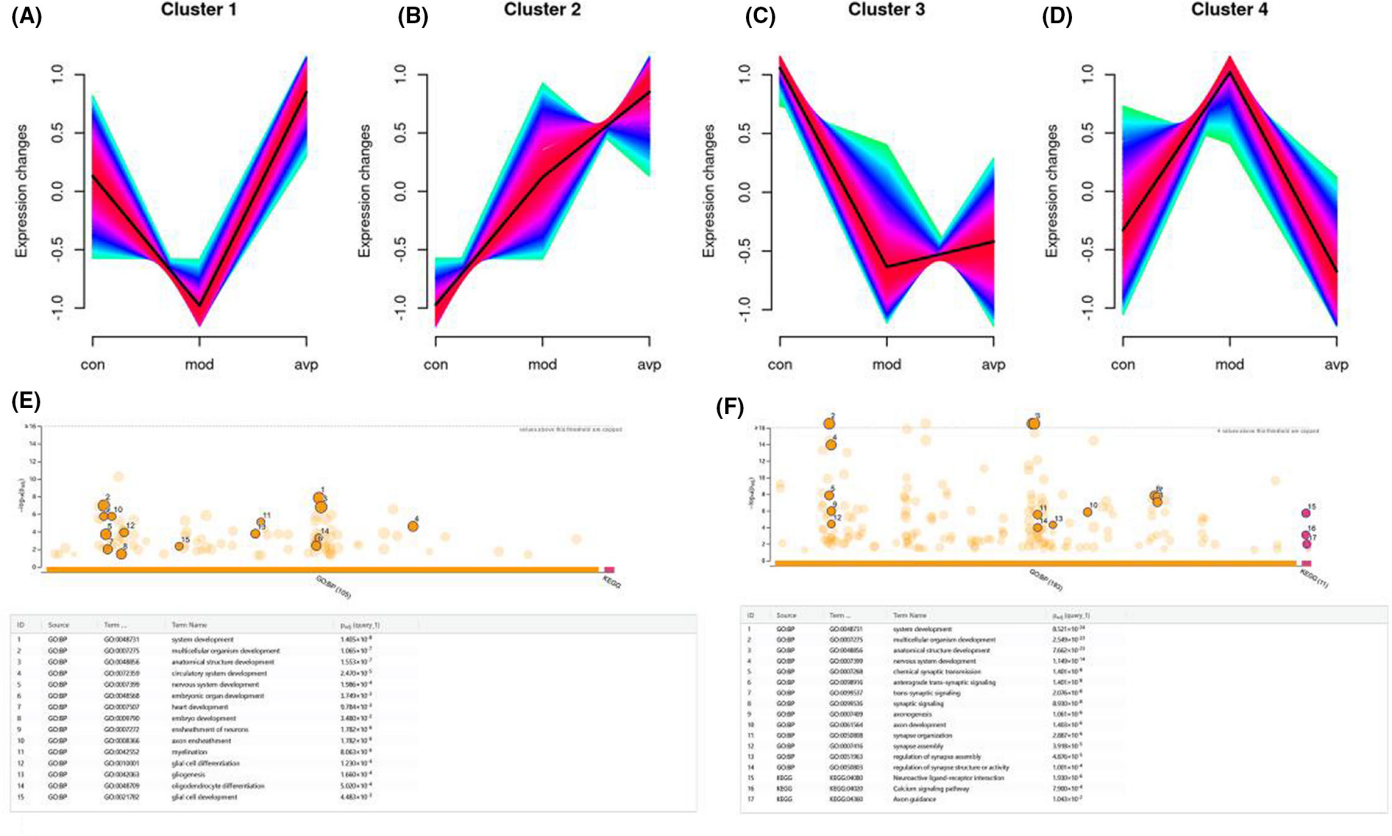
The expression trends of top 3000 most variable genes were analysed using Mfuzz. (A) Clusters 1 presented a trend of decreasing in the VPA‐induced autism model group and increasing in the AVP group, (B) clusters 2 presented a trend of continuous increase in the VPA‐induced autism model group and AVP group, (C) Clusters 3 presented a trend of decreased in the VPA‐induced autism model group and AVP group, (D) Clusters 4 presented a trend of increasing in the VPA‐induced autism model group and decreasing in the AVP group, (E) Function annotation of genes in Clusters 1, (F) Function annotation of genes in Clusters 4.

The expression trends of top 3000 most variable genes were analysed using Mfuzz clustering analysis, and 4 clusters with different change trends were screened out (Figure 6A–D). We further analysed the genes biology function in the cluster 1 and cluster 4. Genes in cluster 1 presented a trend of decreasing in VPA‐induced autism model group and increasing in AVP group (Figure 6A). A large number of organ development (system development, circulatory system development, nervous system development, heart development, embryo development and so on) and neuroglia development (gliogenesis, glial cell development and differentiation, oligodendrocyte differentiation, myelination and so on) related biological processes are enriched, no signal pathway were enriched in this cluster (Figure 6E). Genes in cluster 4 presented a trend of increasing in VPA‐induced autism model group and decreasing in AVP group (Figure 6D). lots of genes were enriched in organ development (system development, multicellular organism development, nervous system development and so on) and synaptic development (chemical synaptic transmission, anterograde trans‐synaptic signalling, trans‐synaptic signalling, synapse assembly and so on) related biological processes, and these gene were mainly involved in neuroactive ligand–receptor interaction, calcium signalling pathway and axon guidance (Figure 6F).

### 3.5 RNA‐Seq results were validated by qPCR

**FIGURE 7:**
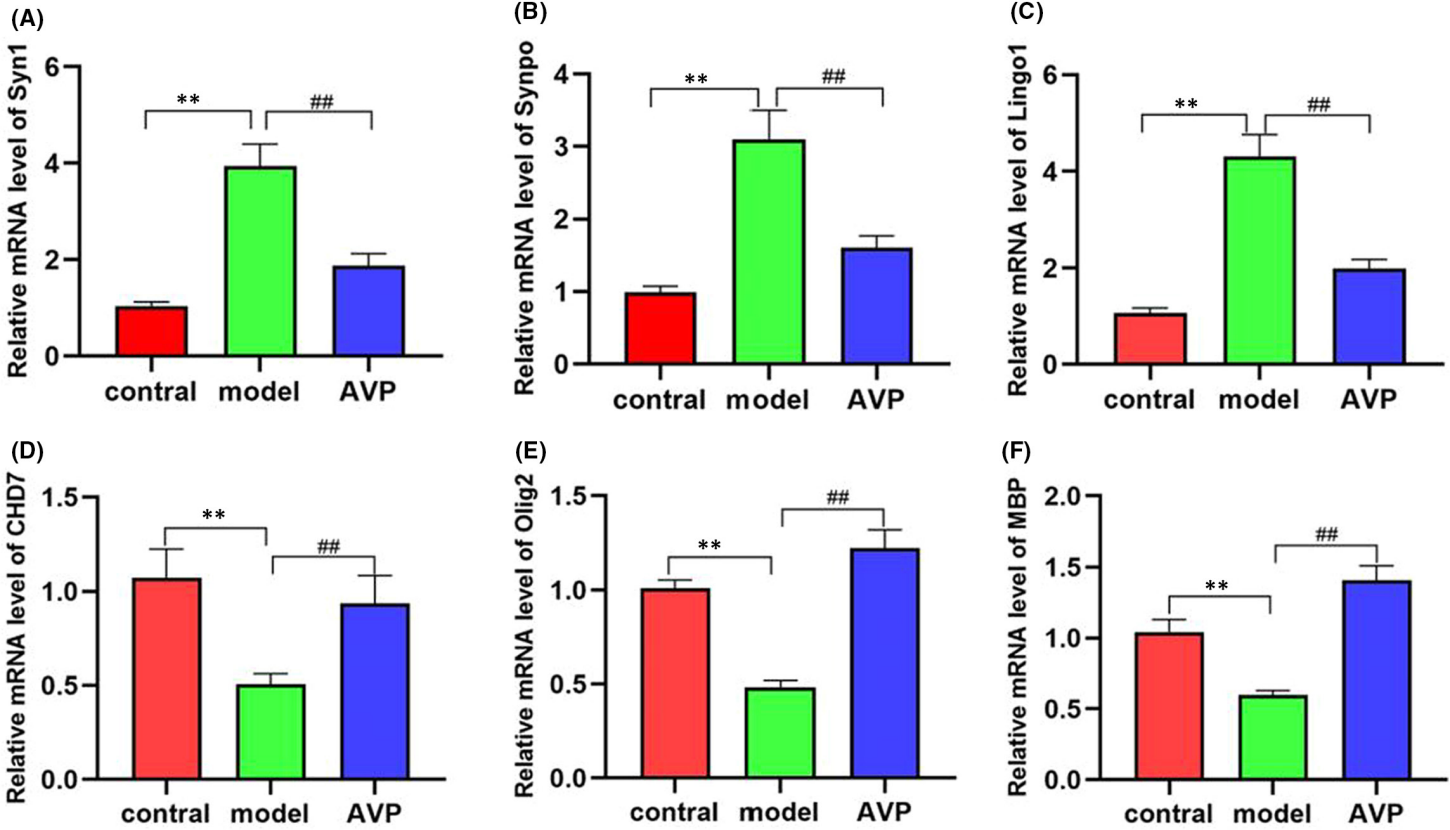
The mRNA levels of synaptic and myelin development related genes (*n* = 5). Compared with the control group: **p < 0.01, compared with the VPA‐induced autism model group: ^##^ *p* < 0.01.

To confirm the RNA‐seq data, we performed qPCR to examine the expression levels of selected synaptic and myelin development related genes (Figure 7). Compared with the control group, the mRNA levels of synaptic development‐related genes SYN1, SYNPO, and axon development‐related genes Lingo‐1 were significantly increased in the VPA‐induced autism model group (*p* < 0.01) and improved after AVP treatment (*p* < 0.01). In addition, the mRNA levels of brain development‐related genes CHD7 and oligodendrocyte development‐related genes Olig2 and MBP were significantly decreased in the VPA‐induced autism model group (*p* < 0.01), and improved after AVP treatment (*p* < 0.01).

## 4 DISCUSSION

We have demonstrated that AVP can significantly improve social dysfunction in VPA‐induced rats with autism in previous studies. To further explore the mechanisms of action of AVP, we analysed and compared the transcriptomic changes occurring in the PFC of ASD model with or without AVP treatment using DEG, GSEA and Dynamic expression patterns analyses in the present study.

### 4.1 AVP significantly ameliorates synaptopathy in autistic rat model

Synapses are essential components of neurons and allow information to travel coordinately throughout the nervous system to adjust behaviour to environmental stimuli and to control body functions, memories and emotions.^32^ Increasing evidence demonstrates the importance of synapse dysfunction as a major determinant of several neurodevelopmental diseases, such as ASD.^33^, ^34^, ^35^ Our result shows that synaptic development‐related biological processes such as chemical synaptic transmission, anterograde trans‐synaptic signalling, trans‐synaptic signalling, synapse assembly and so on were significantly enhanced in the autism model group and significantly decreased after arginine treatment. This result is consistent with the transcriptome study of Zhang et al.^36^, ^37^ Subsequently, we confirmed that synaptic development‐related genes SYN1 (Synapsin 1, a neuronal phosphoprotein involving in regulating axonogenesis and synaptogenesis^38^) and SYNPO (synaptopodin, a marker and essential component of the dendritic spines^39^ **)** were significantly upregulated in autism, and significantly improved after AVP treatment. Consistently, Liu et al.^40^ found the Syn1 proteins relevant to synaptic plasticity in the PFC significantly increased in VPA‐induced autism model. Gomes^41^ and Belagodu^42^ et al. also found the Syn1 proteins significantly increased in IL‐17 induced maternal immune activation model and Fragile X Syndrome model.

By pathway enrichment analysis, we found that the neuroactive ligand–receptor interaction and Calcium signalling pathway had the same change trend with synaptic development which significantly increased in the VPA‐induced autism model and decreased after AVP treatment. The neuroactive ligand‐receptor interaction signalling pathway is a collection of receptors and ligands on the plasma membrane that are associated with intracellular and extracellular signalling pathways,^43^, ^44^ and this pathway may influence the physiological and pathological functions of neuronal by ligand‐receptor interactions in autism.^45^, ^46^ Calcium signalling is involved in many essential cellular functions and plays a key role in synaptic plasticity, neuron excitability, axon growth and neurotransmitter release and so on.^47^ Given its physiological importance, abnormalities in neuronal Ca^2+^ signalling potentially underlie many different neurological diseases such as autism.^48^, ^49^, ^50^

### 4.2 AVP significantly ameliorates axonal growth and regeneration inhibition in autistic rat model

Axon guidance and synapse formation are important developmental events for establishing a functional neuronal circuitry.^51^, ^52^ Studies have shown that abnormal axon guidance signalling were associated with brain microstructure abnormalities in autistic patients.^53^, ^54^ Our results showed that axon development‐related biological processes such as axonogenesis, axon extension and axon regeneration and so on were significantly enriched. This is consistent with the transcriptomic analysis of Zhang et al.^55^ Axon growth and regeneration inhibition‐related genes or gene sets were significantly upregulated in autism model, and significantly improved after AVP treatment. Further qPCR confirmed Lingo1 (Leucine rich repeat and immunoglobulin‐like domain‐containing protein 1),^45^, ^46^ a transmembrane signalling protein expressed in both neurons and oligodendrocytes which inhibits oligodendrocyte differentiation, axonal regeneration and myelin production, were significantly upregulated in autism and significantly improved after AVP treatment. The results showed that AVP could significantly ameliorate VPA‐induced axonal growth and regeneration inhibition in prefrontal cortex.

### 4.3 AVP significantly promoted oligodendrocyte development and myelination

Oligodendrocyte are glial cells in the CNS, responsible for myelin sheath formation, which allows fast signal transmission, provides metabolic support to axons.^56^ Deficits in Oligodendrocyte development and function will lead to demyelination and axonal dysregulation, and disruption in neuron–glia interactions promote autistic‐like features.^57^, ^58^ Our results showed that oligodendrocyte development and differentiation and myelination‐related biological processes were showing a downward trend, but not significantly in VPA‐induced autism model group compare to control. However, these biological processes were significantly upregulated after arginine treatment. Dynamic expression patterns analysis also showed genes/gene sets of these biological processes tended to decrease in the VPA‐induced autism model group and increase after AVP treatment. Subsequently, we confirmed that Oligodendrocyte and myelin development related genes Olig2 (a transcription factors necessary for Oligodendrocyte development) and MBP (a structural component of myelin, expressed exclusively by myelinating glia), and brain development key regulatory factors CHD‐7^59^, ^60^, ^61^ (Chromodomain helicase DNA binding proteins 7, which is highly expressed in differentiating Oligodendrocyte) were significantly downregulated in autism, and significantly improved after AVP treatment by qPCR. Obviously, deficits in OL development and function may be one of the core mechanisms of autism.^62^, ^63^

Pathway enrichment analysis showed ribosome, fatty acid metabolism and PPAR signalling pathway had the same change trend with oligodendrocyte development and myelination which significantly reduces in the VPA‐induced autism model and increase after AVP treatment. The results showed that inhibition of protein synthesis and fatty acid metabolism was closely related to the occurrence of autism,^64^, ^65^, ^66^, ^67^, ^68^ which may be related to the inhibition of myelin sheath generation.^69^, ^70^ Upregulation of these signalling pathways may promote glial development and myelination, and improve core symptoms of autism.^71^, ^72^ Recently, a growing body of evidence suggests that PPARs play a key role in the pathophysiology of ASD. Khera et al.^73^ found Guggulsterone significantly improved the neurobehavioral and neurochemical abnormalities in propionic acid‐induced autism model with increased MBP and decreased demyelination by activating the PPARγ signalling pathway. Cristiano et al.^74^ found endogenous lipids palmitoylethanolamide (PEA), a PPAR‐α agonist, reverted the altered behavioural phenotype of BTBR mice, and this effect was contingent to PPAR‐α activation. Obviously, PPAR may be a new therapeutic target for autism.

In summary, our results have revealed AVP can significantly improve synaptic and axon dysplasia and promote oligodendrocyte development in the prefrontal cortex in VPA‐induced autism models by regulating multiple signalling pathways. It provides a mechanistic framework to understand how does arginine vasopressin improve autism social interaction disorder.

## AUTHOR CONTRIBUTIONS

**Bo Zhou:** Investigation (equal); methodology (equal); visualization (equal); writing – review and editing (equal). **xuehui Yan:** Investigation (equal); methodology (equal); validation (equal). **Liu Yang:** Methodology (equal); writing – original draft (equal). **xiaoli Zheng:** Methodology (equal). **yunhua Chen:** Methodology (equal). **yibu Liu:** Investigation (equal). **yibing Ren:** Investigation (equal). **jingang Peng:** Investigation (equal); methodology (equal). **yi Zhang:** Methodology (equal). **jiayu Huang:** Conceptualization (equal). **lei Tang:** Conceptualization (equal). **min Wen:** Conceptualization (equal); writing – review and editing (equal).

## CONFLICT OF INTEREST

The authors confirm that there are no conflicts of interest.

## Data Availability

The data that support the findings of this study are available from the corresponding author upon reasonable request.
